# The cell-mediated adaptive immune response to herpes simplex virus type 1 encephalitis: mechanisms and clinical implications

**DOI:** 10.3389/fimmu.2026.1751672

**Published:** 2026-07-10

**Authors:** Louise Osborne, Cordelia Dunai, Yun Huang, Franklyn N. Egbe, Lance Turtle, Benedict D. Michael, Mark A. Ellul

**Affiliations:** 1 Clinical Infection, Microbiology and Immunology, Institute of Infection, Veterinary and Ecological Sciences, University of Liverpool, Liverpool, United Kingdom; 2 National Institute of Health and Care Research Health Protection Research Unit in Emerging and Zoonotic Infections, University of Liverpool, Liverpool, United Kingdom; 3 The Walton Centre National Health Service (NHS) Foundation Trust, Liverpool, United Kingdom; 4 Tropical and Infectious Diseases Unit, Royal Liverpool University Hospital, Liverpool, United Kingdom

**Keywords:** adaptive immunity, encephalitis, herpes simplex virus, inflammation, T cells

## Abstract

Herpes simplex virus (HSV) encephalitis is the most frequent cause of sporadic encephalitis globally. Despite the emergence of the antiviral drug aciclovir curtailing mortality, this disease remains a clinical challenge due to its rapid progression and associated neurological sequelae; indicating the urgent requirement for adjunctive neuroprotective treatment options. Recent studies using both human samples and murine models have highlighted how cell mediated immune cells including CD8+, CD4+ and brain tissue-resident memory T cells have the capacity to act as a ‘double-edged sword’; both serving to protect the host against HSV encephalitis, but also increasing neuroglial injury by inflammation. Factors which impair cell-mediated immunity may predispose individuals to herpes simplex encephalitis, including defects in viral immune evasion strategies (infected cell protein 47, UL13 kinase), host genetic pre-disposition (toll-like receptor 3 deficiency, lymphotoxin-α deficiency, Rel mutations) and external factors such as stress. Additionally, there is a particular need for clinical vigilance for patients on immunosuppressive treatments which impair cell-mediated immunity, including azathioprine, hydroxychloroquine and methotrexate. Conversely, understanding these molecular mechanisms may provide new insights into the use of immunomodulatory strategies including anakinra, tocilizumab, and the implementation of targeted vaccination. This review summarises the current state of knowledge of how an impaired cell-mediated immune response could promote herpes simplex virus encephalitis, followed by an exploration of the clinical applications and therapeutic interventions which could viably be implemented to ameliorate immune-mediated tissue injury.

## 1 Introduction

Herpes simplex virus type 1 (HSV-1), a neurotropic α-herpesvirus, typically infects 75% of individuals before they reach the age of 18 (1, 2). Most commonly, the virus becomes latent in the trigeminal ganglion, occasionally presenting as mucocutaneous lesions following reactivation (cold sores) (3). The virus can cause encephalitis at the time of primary infection which usually occurs in neonates (4). However, HSV-1 also has the capacity to reactivate later in life, and infiltrate the brain through the trigeminal nerve or olfactory bulb, by retrograde axonal transport, manifesting as herpes-simplex encephalitis (HSE) (5). HSV-1 is recognised as the most common sporadic cause of viral encephalitis worldwide (6). In response to HSV-1 infection, leukocytes from the peripheral circulation migrate to the brain (7). Leukocyte ingress, cytokine release and cellular injury contribute to an increase in blood-brain barrier (BBB) permeability (8). The combination of viral replication and cell lysis, as well as additional cellular injury through immune activation; ultimately culminates in widespread brain injury (9–11). Hallmark clinical features of HSE include: fever, seizures, headache and reduced conscious level result primarily from the subsequent acute inflammation within the temporal lobes and insular cortex of the cerebral hemispheres as well as concomitant necrosis of neurons, oligodendrocytes and astrocytes (12–14).

The incidence of HSE is approximately 2-4 cases per 100,000 individuals per year (1) with age distribution occurring with two bimodal peaks; one of children aged between 6 months to 3 years, typically due to primary infection (15), and one of adults aged over 50 typically due to viral reactivation (16). Implementation of aciclovir has curtailed the mortality associated with HSE from 75% to around 15-20% (17), however, between 40-60% of those who survive experience mild to severe long-term neurological sequelae (18–20).

**Figure 1:**
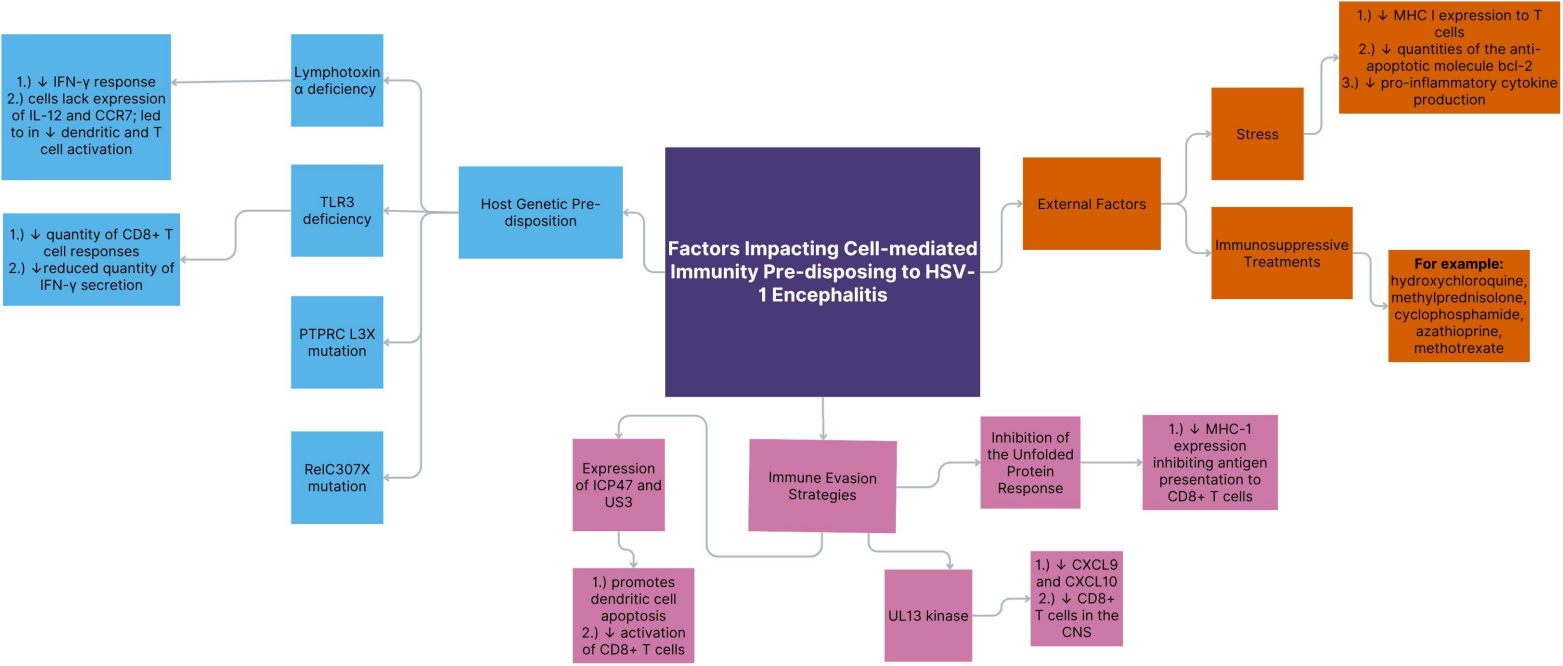
Factors that inhibit cell-mediated immunity and increase the risk of development of HSV-1 encephalitis. IFN-γ, interferon gamma; IL-12, interleukin-12; CCR7, chemokine receptor type 7; TLR3, toll-like receptor 3; ICP47, infected cell protein 47; CXCL9, C-X-C motif chemokine ligand 9; CXCL10, C-X-C motif chemokine ligand 10; MHC-I, major histocompatibility complex class I; bcl-2, B-cell leukaemia/lymphoma 2 protein. Diagram created using canva.com and bioart.com.

Once the virus enters neurons or epithelial cells, pattern recognition receptors (PRRs) are able to detect components of HSV-1 such as envelope proteins and induce the transcription of IFNs and other inflammatory mediators (21). For example, toll-like receptor (TLR) 2 recognizes cell-surface HSV-1 glycoproteins facilitating activation of NF-κB and upregulation of cytokine production (22). Release of cytokines including IL-1β, IL-6 and TNF and chemokines such as CXCL10 and CCL2 promotes recruitment of immune cells including monocytes, natural killer (NK) cells, neutrophils and dendritic cells (DCs) to the site of infection (23). As a result, antigen presenting cells (APCs) such as dendritic cells are able to present viral antigen to CD4+ and CD8+ T cells within secondary lymphoid organs initiating the adaptive immune response (24). The release of pro-inflammatory cytokines may additionally contribute to the changes in BBB permeability which can be seen in the early stages post infection (13, 25). Effector CD8+ T cells are then recruited from the blood into infected tissues by chemokines such as CXCL10, and if the BBB is compromised, also into the CNS parenchyma where HSV-1 viral replication may also occur (26–28). This migration of effector immune cells can either be beneficial to the host through eliminating the cells which have been infected or drive excessive inflammation characteristic of the progression from initial HSV-1 infection to HSE (1). As a result, despite the importance of innate immunity, it is ultimately the adaptive immune response, which occurs through activation of CD8+ and CD4+ T cells and B cells, which may determine whether HSV-1 infection is inhibited, driven into latency or re-activated in the form of HSE (**Figure 1**) (29). It is important to distinguish between humoral and cell-mediated immunity. The adaptive immune response comprises the activation of B and T lymphocytes through both humoral and cell-mediated immunity (30). Cell mediated immunity is independent of antibody production and can facilitate destruction of infected cells via cytotoxic CD8+ T cells or macrophages activated by T helper (Th) 1 cells (a subset of CD4+ T cell) (31). However, there is interaction between both responses as Th1 cells can initiate the production of antibodies capable of opsonisation and a further class of CD4+ T cell, Th2 cells, are able to activate naïve B cells and induce IgM secretion (31).

The neuroglial injury associated with HSE can be attributed to a combination of initial viral replication, an insufficient cell-mediated immune response and an excessive inflammatory response (32). The immunological mechanisms behind why HSV-1 infection manifests as HSE in some individuals but not others is not clear and the relative contribution of T cells has been contested in previous research (33, 34). For example, Zawatzky et al. found that athymic mice without T cells who received intraperitoneal HSV-1 injection were more susceptible to HSE (35) whereas Metcalf et al. found that T cells did not confer any additional protection against HSV-1 infection (36).Differences in viral doses (10^5^ plaque-forming units/ml versus 10^6.8^ TCID_50_/ml by Zawatzky et al) and infection routes (intracranial versus intranasal) could be one explanation for the inconsistent findings (35, 36). Additionally, the strains of mice used by Zawatzky et al. exhibited differences in IFN production compared to other studies as it arose in both the athymic and control conditions (35). An IFN response, initiated after PRR recognition of virus and phosphorylation of transcription factors IRF3 and IRF7, establishes an early anti-viral response and could therefore have conferred protection for these mice (37). Further studies support the role of IFNs, as mice with an insufficient innate antiviral response due to a lack of IFN receptors or STAT1 were more susceptible to HSV-1 infection (38, 39). More translational research is needed to understand the optimal immune response to HSV and this will differ according to kinetics of the infection as well as host factors such as age and immunophenotype.

## 2 Pathophysiology of HSV-1

After primary infection, HSV-1 invades and undergoes replication within epithelial cells of the skin or mucosal surfaces before establishing latency in neuronal cells, with systemic spread of the virus not occurring in those who are immunocompetent (40). Damage to the protective outer layers is typically required to facilitate infiltration of the virus, and the resultant damage to tissues and inflammation can cause the vesicular lesions associated with herpes virus infection (40). After replicating in epithelial cells, the virus travels to the nerve terminals of peripheral neurons and is carried retrogradely towards the neuronal cell body (40). Either lytic or latent replication can then arise, and diminished expression of viral proteins including virion protein 16 (VP16) and infected cell protein 0 (ICP0) may contribute to driving the virus into latency (40). The VP16 promoter consists of binding sites for Egr-1 and Sp1, which are responsive to transcription factors produced following neuronal stress (41). This may increase VP16 transcription and drive reactivation of HSV-1 in neurons (40). In cell culture, stimuli which induce neuronal stress include UV light exposure, histone deacetylase inhibition and inhibition of neurotropic factors whereas in humans, hormonal changes and UV light exposure can trigger transition of the virus from a latent to lytic state (42). The re-activation of HSV-1 and sequelae such as HSE and autoimmune encephalitis are complex processes and are not fully understood.

### 2.1 Herpes simplex encephalitis

In approximately 1-2/10,000 of those infected with HSV-1, the virus invades the CNS either through the olfactory bulb or trigeminal nerve which may eventually progress into either forebrain (95% of cases) or brainstem HSE (5% of cases) respectively (1, 43, 44). The exact mechanism by which HSV-1 infection develops into HSE is uncertain, however a sufficient innate and adaptive immune response may be important in protecting individuals following HSV-1 invasion into the CNS (45–49).

Conversely, an excessive, dysregulated immune response to HSV-1 is implicated in the development of HSE. Signs of intense immune system activity and extensive neuronal destruction in HSE CSF have been observed (50). Molecules which are indicative of immune activation including neopterin and β2-microglobulin, were shown to correlate with mortality and clinical outcome, and were raised during acute onset of disease (51). Additionally, IL-6 and IFN-γ expression is acutely raised, however as infection progresses indicators of cell-mediated immunity including soluble interleukin-2 receptor (sIL-2R) and soluble CD8 antigen (sCD8) become more prominent (51). Simultaneously, chemokine and chemoattractant expression by microglia facilitates the recruitment of peripheral leukocytes (40). Activation of T cells increases and intrathecal immune activity remains high for prolonged periods in many patients with HSE (51), suggesting the existence of an excessive neuroinflammatory response forming part of the pathogenesis of the disease. This inflammation may contribute to BBB changes observed in patients with HSE including vascular brain oedema and haemorrhage, potentially resulting in neurological sequelae such as behavioural change, reduced levels of consciousness and cognitive decline (40, 52).

The interplay between HSV-1 and the host immune system is important in determining outcomes post infection (40). Prevalence of HSE is high in children and the elderly, with 30% of cases arising in those aged between 3 months and 6 years old (53, 54). This may be due to impaired cognitive reserve, or immune senescence which may reduce efficacy of the anti-viral defence usually co-ordinated by IFNs such as IFN-α/β and IFN-λ (54, 55). Linked to this, approximately 5% of individuals who develop HSE have a rare single-genic (monogenic) immune defect (56). HSE incidence has not been shown to be higher in those with genetic defects in leukocytes (54, 57), however deficiencies in genes which co-ordinate the immune response to HSV-1 such as TLR-3 deficiency and the MHC-I allotype result in a predisposition to the disease (58). In these cases, inborn genetic defects have been implicated in 7% of childhood encephalitis cases with insufficient CNS TLR-3 and type I IFN responses described in approximately 5% of children who develop HSE (54, 59).

### 2.2 Autoimmune encephalitis

The development of HSE following HSV-1 infection has been infrequently reported as a trigger of autoimmune encephalitis and the pathogenesis for this is unclear (60). Despite autoantibody production being observed in ~25% of patients with HSE (21), future research should target understanding the interaction between the cell-mediated and humoral immune responses and how this is linked to the onset of disease. It has been proposed that the extensive neuronal damage and inflammation seen in HSE results in a loss of self-tolerance, leading to the generation of antibody against CNS components including the N-methyl-D-aspartate (NMDAR) and dopamine-2 receptors (61, 62). 20% of patients who have had HSE develop antibodies against the N-methyl-D-aspartate receptor (NMDAR), however this only manifests into anti-NMDAR encephalitis in a minority of patients (21, 62). Confirming this, a recent systematic review and meta-analysis found that 89.3% of patients with autoimmune encephalitis were positive for NMDAR antibodies (55). Alongside this, age-dependent differences in clinical presentations could be identified (55). For example headaches and neurobehavioral changes were more frequently reported in those > 12 years of age, whereas seizures and movement disorders were more common in those < 12 years of age (55). Further features observed which were central to each age group included a new brain necrosis found on MRI in those aged 0-12 years, compared to encephalopathy, raised CSF protein and speech dysfunction found in the > 12 year cohort (55). The retrospective nature of these reports may have made them subject to bias, however sensitivity analysis which was conducted suggested that bias did not change the reports of age-specific clinical features which were observed (55). The clinical benefit associated with the use of immunotherapies was also difficult to establish, as it was delivered in 90.7% of cases and reports provided no indication of timing of implementation (55). As a result, studies using global data-sets that can inform whether immunotherapy implementation determines outcome are required (55).

### 2.3 Alzheimer’s disease

HSV-1 was proposed as a possible aetiology of Alzheimer’s disease (AD) following observations that the temporal lobe, frontal lobe and hippocampus were affected in both severe HSE and AD (63, 64). Despite the fundamental differences between HSE and AD, some overlapping symptoms are present including headaches and seizures (65). A retrospective cohort study conducted over 10 years recently found a 2.56 fold increased risk of all types of dementia in patients with a history of HSV-1 infection (66). This study matched a large sample of 25,086 subjects increasing representation, however a prospective longitudinal cohort study is required to allow for an accurate sequence of events to be determined and risk of recall bias to be reduced (66).

Latent HSV-1 viral DNA was initially localised to the temporal and frontal lobes using polymerase chain reaction (67). Importantly, these samples were taken from autopsies, increasing the risk of postmortem changes to the tissue integrity limiting the accuracy of the biomarkers (67). Additionally, HSV-1 DNA is also found in a large proportion of elderly control brains, which can be attributed to the fact that HSV-1 seroprevalence has been reported to be as high as 63.5% among adults of the general population (63, 68). HSV-1, when combined with the presence of the apolipoprotein E (APOE) gene, has also been found to lead to a higher risk of AD (69). However, AD has a complex aetiology which is not just restricted to either the presence of HSV-1 DNA or the APOE gene (63). This is demonstrated by the finding that 60% of patients with AD were found to have a combination of both a viral and genetic predisposition, however in the other 40% of AD patients the factors contributing to the onset of pathology were not clear (63). However, in a study which analysed 439 brain specimens of patients with AD (APOE positive) for the DNA of HSV-1 found there was no increased risk of AD in patients infected with the virus (70). A further study assessing the association between HSV-1, the APOE gene and AD based on autopsies and subsequent PCR analysis of DNA found no association (71). As a result, the evidence supporting the association between HSV-1, AD and the APOE gene is mixed and further studies clarifying the relationship between the variables is required (63, 70, 71).

Proposed mechanisms for the pathogenesis of AD include β -amyloid (Aβ) accumulation, hyperphosphorylation of tau protein, mitochondrial dysfunction and oxidative stress (72). There is evidence suggesting that HSV-1 re-activation may contribute to changes in the amyloid precursor protein (APP) and increase expression of Aβ (73). Previous research has localised HSV-1 viral DNA to Aβ plaques using brain specimens from 6 patients with AD and 5 controls (74). However, it is important to consider both the small sample size and the possibility that other factors may have caused the viral DNA to be present within the plaques such as the migration of microglial cells (74). A further study found that HSV-1 infection of neuroblastoma cells led to a marked increase in expression of a 55kDa C-terminal APP fragment (73). However, infection of brain endothelial cells with *Chlamydia pneumoniae* increased the intensity of a similar APP fragment, indicating that this fragment may represent a typical stress response to infection (73). Further observations reported that HSV-1 binding to rat neurons induced depolarization of the membrane, resulting in increased intracellular Ca2+ signalling which was able to induce phosphorylation and later cleavage of APP through the action of glycogen synthase kinase 3 (GSK3) (75, 76). *In vitro* studies have also attributed GSK3 to the hyperphosphorylation of tau during HSV-1 infection (77). HSV-1 has been found to induce the accumulation of Aβ in mouse cortical neurons resulting in synaptic dysfunction (76). In this case, induced HSV-1 re-activations in mice were found to lead to both hyperphosphorylated tau and Aβ within the hippocampus and neocortex (78). Additionally, the quantity of Aβ accumulation following repeated HSV-1 re-activations was found to be associated with the deficits in cognition observed in infected mice (78).

Importantly, most of the research supporting an association between HSV-1 and AD is cross-sectional or observational which is therefore unable to completely exclude confounding factors (such as timing of infection, host genetics, immunological factors) or bias (66, 79). As a result, prospective randomised trials which determine whether antiviral or vaccination strategies lower the incidence of AD are required to inform future management (66, 79). Furthermore, future research should look to determine how the quantity and timing of viral dissemination to the CNS correlates with risk of AD to address gaps in the research and subsequently use this to inform viable ways we can prevent the development of AD (72).

## 3 Components of the cell-mediated response to HSV-1 in the central nervous system

The antiviral response to primary HSV-1 infection involves a combination of both innate and adaptive immunity and is initiated following TLR-dependent detection of HSV-1 by TLR2, TLR3 and TLR9 (29, 80). TLR2 is able to recognise HSV-1 glycoproteins on the cell surface, whereas TLR9 senses viral DNA, both of which then activating cGAS and adaptor proteins such as STING (81). Activation of the innate immune response is also co-ordinated by retinoic acid-inducible gene I (RIG-I)-like helicases (RLRs) can recognise cytoplasmic HSV-1 RNA within infected cells initiating downstream signalling including NF-κB, activator protein 1 (AP-1) IFN regulatory factors (IRFs) including transcription factors such as IRF3 and IRF7 (82). These transcription factors co-ordinate type I IFN and proinflammatory cytokine production which help to initiate the cell-mediate immune response to HSV-1 infection (81, 83). Each cell type of the cell-mediated response to HSV-1 infection responds uniquely to help contain the infection, with an insufficient response potentially contributing to the development of HSE (21).

### 3.1 Antigen presenting cells

APCs including DCs and macrophages are sentinel immune cells which can present antigen to T cells as small peptides loaded on MHC-I and MHC-II following detection of HSV-1 (84). DCs detect HSV-1 via expression of a vast array of intracellular and extracellular PRRs (85, 86) and take up antigen via unique receptors including endocytic and C-type lectin receptors (87). One study found that *in vitro* treatment of bone marrow DCs with TLR2 increased secretion of IL-6 and IL-12 (88). Release of these cytokines was regulated by the stimulation of both TLR2 via viral glycoproteins such as gH (89) and TLR9 due to the presence of endosomal viral DNA (88). Detection of HSV-1 by DCs can then promote secretion of IFN- α and IFN- β, which attempts to control HSV-1 by inhibiting spread of infection from the periphery to the CNS and inhibiting expression of HSV-1 genes (90). DCs are then able to transport antigen to lymph nodes where the peptide fragments can be presented to and contribute to the activation of CD4+ and CD8+ T cells and allow them to exert their effector functions (87, 91). Depletion of DCs from mice who had undergone ocular infection with HSV-1 was found to inhibit viral clearance due to the reduction in chemokines including CCL5, CCL7, CXCL9 and CXCL10 (92).

Macrophages contribute to the immune response to HSV-1 by both acting as APCs and contributing to pro-inflammatory cytokine production (93). Macrophages facilitate IL-12 production (94, 95), which promotes the differentiation of Th1 cells and secretion of IFN-γ and TNF (96, 97). Supporting this, the treatment of mice with TNF conferred protection against intraperitoneal HSV-1 infection (98). Macrophages sub-types can be classified into M1 and M2, with M1 macrophages producing a larger number of proinflammatory cytokines such as IL-6, IL-12, TNF and IFN-γ alongside producing reactive oxygen species via the action of inducible nitric oxide synthase (99, 100). M2 macrophages, which are induced by IL-4, IL-13, IL-10 or glucocorticoids, instead produce immune mediators which are anti-inflammatory such as IL-10, TGF-β and arginase 1 (99, 101). One study found that M1 macrophages either derived from RAW264.7 cells or C57BL/6 mice produced more proinflammatory immune mediators compared to M2 macrophages (93). However, reduced viral replication was observed in M1 macrophages studied *in vitro* compared to those *in vivo* indicating that the *in vitro* studies may not have been representative of the tissue specific effects and other immune cells which may influence the immune response to HSV-1 (93). Despite these limitations, studies such as these lend support to the notion that M1 macrophages may predominantly exert anti-viral action whereas M2 macrophages may reduce inflammation (102, 103). As a result, research has been undertaken to determine whether polarisation of M2 macrophages from the M1 sub-type could be used therapeutically in the management of herpetic stromal keratitis, which is a complication of recurrent ocular infection with HSV-1 which could potentially lead to blindness (104, 105). Supporting this, the Ghiasi group have found that polarization to M2 macrophages using IL-4 administration in mice reduced the inflammatory response following ocular HSV-1 infection (106). However, research is conflicting as M2 polarization has also been demonstrated to increase both primary and latent HSV-1 infection in mice due to increased phagocytosis (107). Therefore, despite this therapeutic promise more studies are required to clarify these gaps in knowledge and determine whether polarization to M2 macrophages could be used to manage complications of recurrent ocular HSV-1 infection such as HSK (104). Alongside facilitating innate immunity, macrophages phagocytose viral antigens and act as APCs (108). Following phagocytosis, they express B7 and MHC-II which can activate T cells via co-stimulation signalling (108). Simultaneously, macrophages co-ordinate the ubiquitination of the viral capsid facilitating its proteasomal degradation and exposure of viral DNA (109).

### 3.2 Neutrophils

Neutrophils have been reported as the first leukocyte subset to arrive to areas of viral infection (110), however there has been conflicting research which found no evidence of neutrophil recruitment following inoculation of a mouse footpad model (111). Opsonisation of microbes via Fc receptors initiates the phagocytic properties of neutrophils (110). Neutrophil recruitment into the CNS of a mouse model of HSE has been found to drive morbidity (112). In this model, cessation of CXCL1 release using Cxcr2-/- mice markedly diminished neutrophil migration and BBB permeability (112). Morbidity of these mice was also substantially reduced despite no change in viral load (112). Intravital microscopy also revealed that CXCR2 is a mediator of neutrophil migration, identifying the CXCL1-CXCR2 axis as a potential promoter of the BBB breakdown seen in HSE (112). This BBB injury may be associated with retention of neutrophils within the perivenular space and further insights are required to determine the precise way that neutrophils may promote BBB breakdown (112). However, *in vivo* studies have associated migration of neutrophils with mechanisms including protease release and diminished tight junction integrity (113, 114).

Neutrophil accumulation in the corneal stroma in mice with ocular HSV-1 infection was associated with the release of further proteases and cytokines, alongside inducible nitric oxide synthase and reactive oxygen species (115). Furthermore, BALB/c mice treated with the antibody RB6-8C5 which depleted neutrophils were more susceptible to viral spread to the skin and brains, increasing susceptibility to HSE (116). This suggests that neutrophils are important in preventing the spread of HSV-1 post ocular infection (116). However, it is important to consider that there was no significant difference in HSK severity between the treated mice and the controls, indicating that HSK is not predominantly driven by neutrophils and instead the contributing roles of other effector cells including macrophages and T cells must also be appreciated (116).

### 3.3 Microglia

**Figure 2:**
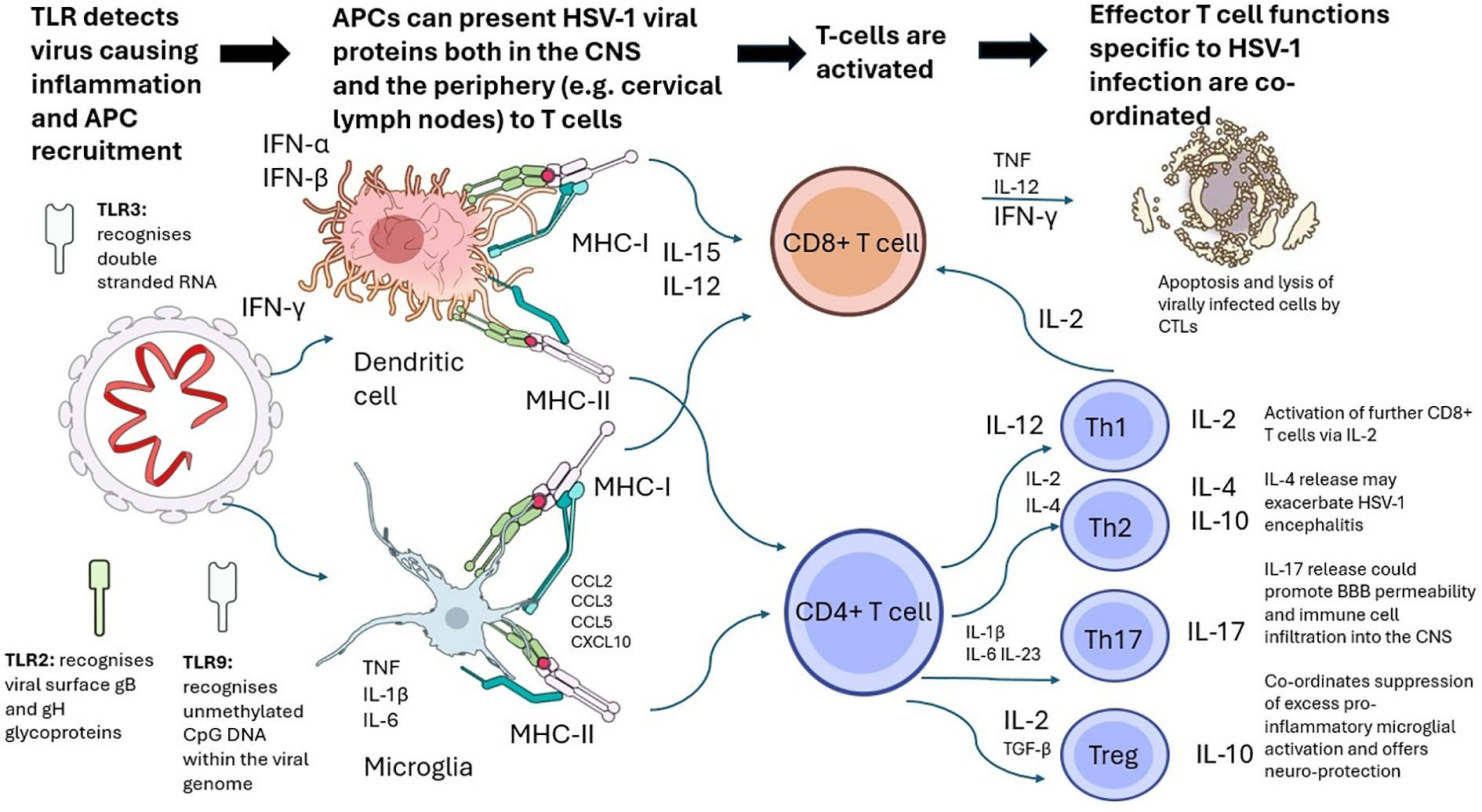
Immune response to HSV-1 encephalitis in the CNS and the periphery. IFN-γ, interferon gamma; IFN-α, interferon alpha; IFN-β, interferon beta; MHC-I, major histocompatibility complex type I; IL-15, interleukin 15; IL-12, interleukin-12; TNF, tumour necrosis factor; IL-1β, interleukin-1 beta; IL-6, interleukin-6; MHC-II, major histocompatibility complex type II; CCL2, C-C motif chemokine ligand 2; CCL3, C-C motif chemokine ligand 3; CCL5, C-C motif chemokine ligand 5; CXCL10, C-X-C motif chemokine ligand 10; IL-2, interleukin-2; TGF-β, transforming growth factor-beta; IL-23, interleukin-23; IL-4, interleukin-4; IL-10, interleukin-10; IL-17, interleukin-17; PRR, pattern recognition receptor; TLR2, toll-like receptor 2; TLR9, toll-like receptor 9. Diagram created using bioart.com.

Microglia serve as the resident immune cells of the CNS (117). They detect viral pathogens through pattern recognition receptors and initiate antiviral responses through proinflammatory cytokine production (**Figure 2**) (118). They also have capacity to phagocytose, present antigen to T cells and drive leukocyte recruitment alongside being a predominant source of type I IFN (119, 120). A recent study found that single-cell RNA sequencing of isolated cells from the brainstems of mice with corneal HSV-1 infection led to strong immunological gene expression by CD45+ cells (121). Dominant cell types were microglia and monocytes followed by T cells in later stages of infection (121). Increased T cell expression continued even after viral clearance. Linked to this, depletion of microglia has been found to diminish IFNβ which could impair T cell activation (122–124). Microglia are a predominant source of HSV-induced chemokines including TNF, IL-1β, CCL5, CXCL10 and RANTES (125). Following invasion of HSV-1 into the CNS, resident microglia forgo their previous surveillance role to initiate an immune response via TNF synthesis (122, 126). TNF then proceeds to regulate both the innate and adaptive immune responses through autocrine and paracrine mechanisms of action (32). As a result, this inflammatory response is essential in orchestrating the rest of the cell-mediated response (119). Sustained increases in TNF were observed in 9 serum and CSF samples from patients with acute HSE, indicating that an excess of this cytokine may contribute to the brain injury seen in HSE (127, 128). In a murine model of HSE, combined treatment of mice with valaciclovir and etanercept (a soluble dimerised TNF receptor) led to increased survival compared to those who received antiviral alone (129), indicating that the increase in TNF production mediated by microglia could be targeted therapeutically (129). Microglia additionally respond to IFN-α production by upregulating TLRs (TLR3, TLR4, TLR7) on DCs and lymphocytes, which further increases IFN and cytokine release (130). The cytokines TNF and IL-1β, secreted by microglia, have also been reported to exhibit neurotoxicity (125). Synthesis of reactive oxygen species (ROS) by microglial cells infected with HSV-1 may also lead to brain damage via the production of nitric oxide synthase and cytotoxic nitric oxide (131). The infection of murine microglial-neuronal cell cultures with HSV-1 increased ROS levels, inducing a redox imbalance which increased lipid peroxidation and oxidative stress (132). Oxidative stress is also capable of enhancing Aβ accumulation and impairing autophagy – mechanisms which could be implicated in the pathogenesis of AD (132).

Experimental models using HSV-1 infected brains of mouse models and HSE patients have revealed apoptosis in microglia is mediated by the DNA-sensing cGAS/STING pathway which also induces IFN I signalling (120, 133, 134). IFN I, TNF and IL-6 production is also initiated by microglial recognition of TLR3 (135–137). Emerging research has found that the Golgi apparatus (GA) may contribute to TLR and cGAS/STING signalling (138). The golgi matrix protein 130 (GM130) promotes GA structural integrity alongside regulating post-translational glycosylation of proteins such as TLR3 (139, 140). Experiments using *in vivo* HSV-1 infected BV2 cells derived from mice found that GA stress mediated by GM130 downregulated TLR3 expression by microglia (141). It also reduced IFN-β, TNF and IL-6 expression which may enhance HSV-1 viral replication and susceptibility to CNS invasion (141).

Similarly to macrophages, microglia have the capacity to polarise into either a pro-inflammatory (M1) or anti-inflammatory (M2) type (72). Persistent overactivation of microglia during either late lytic or latent infection may drive an excessive pro-inflammatory M1 response, characterised by secretion of cytokines such as IL-1β, IL-6 and TNF (72, 142). One study using mouse microglial cells found that the gut metabolite nicotinamide n-oxide promotes microglial polarization to the M2 type and inhibits neuroinflammation via activation of SIRT1-mediated p65 deacetylation (143). Factors generated by microglia including the APP and RNAase L can initiate cell death and apoptosis of neurons (144, 145). Exposure of mouse hippocampal slice cultures to lipopolysaccharides (LPS) and IFN-γ induced massive neuronal death and dysfunction, but only when added together (146). This indicates that co-activation via a pathogen-like stimulus and cytokines may be required to induce neurotoxicity (146) The implications of microglial induced neurotoxicity on neurodegenerative diseases is still to be fully elucidated.

Uncontrolled production of pro-inflammatory cytokines including type I IFN, IL-6, IL-1β, IFN-γ and TNF may induce the BBB damage seen in HSE (24). *In vivo* studies using murine brainstems indicate that CD4+, CD8+ T cells and macrophages accumulate next to cells infected with HSV-1, which may increase the extent of neuronal damage and inflammation (147). Leukocyte CNS infiltration is modulated by adhesion molecules (including VE-cadherin, CD99, JAM-A and PECAM), chemokines (CCL5, CXCL10 and CXCL8) and matrix metalloproteinases (MMPs) (148–151). MMP2 and MMP9 are thought to contribute to the BBB dysfunction which is seen in HSE (152). Alongside this, HSV-1 likely causes BBB damage through inflammatory-induced activation and infecting endothelial cells directly (153–155). ROS produced by microglia could increase BBB permeability by contributing to degradation of tight junction proteins (156).

### 3.4 Astrocytes

The role of astrocytes in the immune response to HSV-1 is not completely understood, however their role in upregulating TLRs (TLR2, TLR6, TLR7, TLR8 and TLR9) has been reported (157). The viral protein interferon-inducible protein 204 (IFI204) which usually senses nuclear DNA is also downregulated by astrocytes, which could serve as an immune evasion strategy (82). Astrocytes respond to HSV-1 infection with the expression of type I and type II IFNs, initiating the JAK-STAT pathway (82). STAT4 expression can induce the proinflammatory IL-12 cascade, however unlike microglial cells, astrocytes have not yet been found to produce substances that induce neuronal damage (9, 82). Type III IFNs, also known as interferon lambda (IFN-λ), harness the same signalling pathways as IFN I and have been shown to inhibit DNA replication in astrocytes infected *in vitro* with HSV-1 (158). This has been proposed to arise through the induction of the type I IFN, IRF7 and JAK-STAT pathways (158). However, one study found that following vaginal infection of mice with HSV-2, IFN-λ provided anti-viral protection when stimulated by TLR3 and TLR9 however not when controlling TLR-independent infection (159). Differences in methodology may account for this discrepancy. For example, Li et al. (158) utilised primary human astrocytes which were infected *in vitro* with HSV-1, whereas Ank et al. (159) utilised IL-28Rα-/- and IFNAR-/- mice. As a result, differences in observed roles of IFN-λ in cell culture could be due to the measured responses in cell signalling being based on isolated cells alone. Alongside this, a 20μl of a lethal dose of HSV-2 was delivered vaginally to the mice, which may not be comparable to the MOI dose of 0.01 which was used to infect astrocytes *in vitro* (158, 159). As a result, the inconsistent findings could be attributed to differences in doses, strains of virus used and mechanism of infection. More comparable studies detailing the role of astrocytes following HSV-1 infection are therefore required to obtain a more comprehensive understanding overall.

### 3.5 CD8+ T cells

CD8+ T cells recognise viral peptide presented on MHC class I molecules and directly kill infected cells through the release of perforin and granzyme (160, 161). They also suppress viral replication and enhance immune cell activation through the secretion of cytokines such as IFN-gamma (162, 163). HSV-1 impairs this response through expression of ICP47, which is an immediate-early protein that resists the hosts ability to activate CD8+ T cells (164). ICP47 expression prevents migration of antigenic peptides, leading to the build-up of MHC-I inside the endoplasmic reticulum (ER) (21). This occurs due to ICP47 binding to the transporter associated with antigen processes (TAP) 1 and TAP2, competing with antigenic peptides (165–167). As a result, peptides cannot be transported from the ER to the cytosol by TAP1/TAP2, meaning MHC-I molecules remain in the ER not loaded with peptide (165). If the host can circumnavigate this resistance created by MHC-evasion strategies unique to the virus, antigen can then be presented via MHC-I, leading to the differentiation of naive CD8+ T cells into T cytotoxic (Tc) 1, Tc2 or Tc17 cells (168–170).

Effector CD8+ T cells recognise MHC-I on infected cells and form an immunological synapse, where localised delivery of perforin and granzymes to the target cell can be delivered (171). Perforin facilitates pore formation in the cell membrane and allows for the delivery of granzymes, such as granzyme B, into the cytosol (172, 173). Granzyme B, acting via either a caspase-dependent or independent mechanism can trigger apoptosis and potentially limit viral replication and spread (174, 175). These molecules also confer the usual host immune response to HSV-1 infection through causing DNA fragmentation and loss of membrane integrity, facilitating subsequent viral clearance (176, 177). In sites of peripheral HSV-1 infection such as the cornea and trigeminal ganglia, CD8+ T cells and IFNγ expression could suppress re-activation and infectious spread (178, 179). In the CNS, timely effector CD8+ infiltration could therefore be a factor important in the prevention of uncontrolled viral dissemination and spread, increasing the risk that infection may progress to HSE if this is inadequate (179). This proposed contribution to host protection against HSE by CD8+ T cells provides a feasible explanation for why mice who had insufficient, delayed infiltration of CD8+ T cells into the CNS following HSV-1 infection either intranasally (180) or of the flank (33) had greater susceptibility to developing HSE (33, 180).

CD8+ T cells are able to differentiate into tissue-resident memory T (Trm) cells, relocating from lymphoid tissue into the neural ganglia as this process occurs in mice cutaneously infected with HSV-1 (181). The recruitment of Trm cells into the brain can arise following secretion of IL-1, IL-6, IL-12, TNF and increased MHC expression (182, 183). Roles of these cells include containing infection, contributing to local inflammation, facilitating T cell activation and assisting with antigen presentation (29). Trm within the brain, referred to as bTrm, may confer the ability to halt overactivation of the immune system through promotion of programmed cell death protein 1 (PD-1) synthesis, which acts as an immune checkpoint (184). CD8+ Trm cells also have the capacity to express IFN-γ and granzyme B (185, 186), mediators which have separate roles in co-ordination of the cell-mediated response to initial HSV-1 infection including downstream immune cell activation and cytotoxic activity respectively (185, 186). Intracerebral infection of genetically-modified mice with lymphocytic choriomeningitis virus involved the expression of IFN-γ and granzyme B which induced cytokine release capable of killing virally infected cells (187). In this study, immune cells in the periphery including CD4+, CD8+ and NK cells were depleted using antibodies, which was then confirmed with flow cytometry to ensure that bTrm cells only were being studied (187). However, this model does not account for CNS invasion following systemic infection meaning findings may not be applicable to HSVE (187). Corneal infection of B6 mice with HSV-1 was able to isolate CD4 and CD8 Trm cells in the epithelium of infected cornea (188), which is significant as HSV-1 can travel anterogradely to the corneal epithelium after reactivation (189). Additionally, a reduced number of Trm cells was observed in mice who developed HSK compared to those who did not, indicating Trm cells may provide protection against re-infection with HSV-1 (188, 190). However, the precise roles of each sub-set of memory T cell (tissue-resident, recirculating and memory) in preventing the re-activation of HSV-1 and its role in the development of HSE is still elusive, indicating the requirement for research which clarifies their immunological roles (191).

### 3.6 CD4+ T cells

CD4+ T cells have been demonstrated in mouse models to invade the cornea 4-6 days following ocular HSV-1 infection, with CXCL9 and CXCL10 release promoting this migration (192–195). CD4+ T cells contribute to antiviral immunity by providing help to B cells and CD8+ T cells through antigen presentation and cytokine secretion, including IL-2 and IFN-gamma (29). Naive CD4+ T cells have differentiation capacity to form either T helper (Th) 1, Th17, Th2 or regulatory T (Treg) cells (see **Table 1**) through their interaction with the APC and also the cytokines present in the immunological microenvironment (196, 197).

**Table 1:** The mechanism of activation, proposed contribution to HSV-1 encephalitis and supporting evidence for the roles of different subsets of CD4+ T helper cells.

Subset of CD4+ T helper cell	Mechanism of activation	Proposed contribution to HSV-1 encephalitis	Evidence for this
Th1	- Antigen presenting cells (APCs) secrete IL-12 which stimulates expression of STAT4​ (473, 474) - IFN-γ, synthesized by T cells and NK cells activates STAT1​ (473, 474) - STAT1 and STAT4 combined work to induce expression of the master transcription factor for Th1 cells, T-bet (473, 474)	- Th1 cells co-ordinate IFN- γ secretion, allowing for further activation of microglia and astrocytes which promote MHC class I and II expression, facilitating clearance of viral load (21) - IFN- γ may support elimination of HSV-1 from brain parenchyma by increasing the capacity of macrophages to undergo phagocytosis within the CNS (21) - Th1 chemokines including CXCl9 and CXCL10 help to contain infection through attraction of entry of CXCR3+ into the CNS (21, 29, 475)	- Mice with an uncontrolled inflammatory response co-ordinated by Th1 cells demonstrated greater severity of encephalitis signs (476)
Th2	- Signals from dendritic cells, including IL-2 and IL-4, lead to naïve CD4 T cells differentiating into Th2 cells​ (477) - IL-2 and IL-4 co-ordinate activation of STAT5 and STAT6; culminating in expression of GATA3, which is the main transcription factor allowing for differentiation of Th2 cells (477)	- Il-4, secreted by Th2 cells, may increase HSV-1 encephalitis severity (205)	- Once stimulated with HSV-1, secretion of IL-4 by Th2 cells within the CNS and cervical lymph nodes increased (205) - Mice administered with IL-4 before and after infection with HSV-1 had increased mortality (205)
Th17	- APCs release cytokines which are pro-inflammatory including IL-1β, IL-6 and IL-23, activating STAT3, which inducing expression of the Th17 transcription factor RORγt (478, 479)	- Th17 cells release IL-17, which can disrupt the tight junctions of the BBB and result in widespread CNS inflammation (206, 480) - IL-17 stimulates increased synthesis of chemokines, such as CXCL1 and CXCL2, and cytokines TNF and IL-6 by microglia and astrocytes, which can enhance neutrophil recruitment (206)	- HSV-1 infected mice with miR-155 knockout, preventing expansion of Th17 cells (481), were protected from severe HSE suggesting the link between Th17 cells and HSE pathogenesis (482)
Treg	- Regulatory T cells (Tregs) develop through a process of selection in the thymus (483) - Cytokines including IL-2 and TGF-β are crucial in the activation of Tregs through activation of STAT5 and SMAD2/3, culminating in Foxp3 expression (484–486)	- Tregs, through IL-10 secretion, may contain encephalitis infection and reduce severity (208)	- Zhao et al. (208) transferred Tregs into mice with artificially induced HSV-1 encephalitis; Tregs were found to protect mice against HSE by by controlling T cell responses - Yu et al. (212) found that HSV-1 re-activation from a latent state was reduced when Tregs were able to reduce the size of CD8+ T cell effector cell responses

Th1 cells synthesise IFN-γ via the activation of a JAK/STAT pathway, which is able to establish an antiviral state through inhibition of transcription factors, cytokine upregulation and apoptosis (198). Consistent with this, IFN-γ is capable of inhibiting reactivation of HSV-1 from latency in trigeminal ganglion cultures without any associated neuronal death indicating its potential neuroprotective effects (199). Furthermore, it may upregulate expression of MHC-I and MHC-II which could increase immune cell recruitment (200) and promote macrophage mediated phagocytosis within the brain parenchyma (21). IFN-γ may also induce keratinocytes to release the CXCL9 and CXCL10 which promotes recruitment of CD8+ T cells to the infection site and facilitate protection against HSE (201, 202). During HSK, which arises following recurrent ocular infection with HSV-1, Th1 cells promote the corneal infiltration of a second surge of neutrophils, as a result of the secretion of IFN-γ and IL-2 (104, 203, 204). Th2 cells predominantly co-ordinate B cell antibody production via the release of cytokines including IL-4, IL-5 and IL-13 (202). IL-4 may increase severity of HSE, with one study finding increased mortality in mice administered with this cytokine before and after intra-nasal infection with HSV-1 (205). Th17 cells release IL-17 and IL-22 which can assist in recruitment of inflammatory cells into the CNS (202). For example, IL-17 promotes synthesis of chemokines (CXCL1, CXCL2) and cytokines (TNF, IL-6) which can enhance neutrophil migration (206).

Treg cells are important in regulating latency and reactivation of HSV-1 within the ganglia, as well as having a potential role in preventing the spread of virus into the CNS (207). Furthermore, Zhao et al. (208) found that the transfer of Tregs into mice with artificially induced HSE reduced the size of effector T cell responses, potentially due to the release of cytokines such as IL-10, IL-35 and TGF- β (209, 210). HSK lesions have been found to be exacerbated by Treg depletion in mice, which may be due to a dampened Th1/Th17 effector response (211). Additionally, an induced stress response in mice using heat and restraint was found to activate Treg cells, resulting in the suppression of CD8+ T cells and reduced immune surveillance (212). Alongside this, intra-ocular infection of mice revealed that Treg cells can also induce latency by regulating HSV-1 specific CD8+ T cells (212). However, FoxP3-eGFP was used to indirectly detect the presence of Treg cells and the natural course of HSV-1 infection will differ from the direct intra-ocular infection which was carried out within this study (212). Additionally, alterations in either CD8+ T cells and Tregs alone without changing the other changed whether the virus was driven to latency, therefore further study is necessary to determine the relative contributions of Treg cells, CD8+ T cells and the cytokines they secrete in the induction of latency (212).

### 3.7 B cells

Although secondary autoantibody production arises in roughly one quarter of patients with HSE, knowledge of B-cell responses in acute HSE remain poorly understood (21). However, patients with disorders which reduce antibody production such as combined variable immunodeficiency (CVID) have been reported to develop HSE when not administered intravenous immunoglobulins suggesting the importance of the humoral response (213). Following initial HSV-1 infection, naïve B cells are activated within draining lymph nodes, synthesising virus specific antibodies (IgM initially followed by IgG/IgA following class switch) which can facilitate neutralisation of free virions and opsonisation of infected cells (214). If HSV-1 proceeds to enter the CNS through haematogenous spread or axonal transport an additional intrathecal humoral immune response may be triggered, resulting in the secretion of HSV-1 specific IgG into the CSF (21, 215). A case report where peripheral blood mononuclear cells (PBMCs) and CSF cells were sampled from a patient with HSE five days apart revealed a five-fold expansion of B cell clones in CSF however no such expansion was seen in PBMCs (216). This suggests that an intrathecal expansion of B cells may arise in the CNS during HSE (216).

Additionally, despite viral PCR results from patients being negative, IgG specific to HSV-1 has been shown to remain in CSF for several years post HSE, which suggests that B cell activity may continue in the CNS even after the cessation of viral replication (51, 217). Alongside this, a correlation between a higher IgG index and severity of clinical sequelae has been identified using CSF and serum samples from 20 patients diagnosed with HSE (51). As a result, it has been proposed that a combination of both PCR and detection of intrathecal immunoglobulin may help reduce the false negatives which may arise with PCR-based diagnosis and provide a predictor of prognosis (217). A more recent study found the IgG index of 7 patients with HSV-1 remained significantly elevated after PCR-negative results, further supporting that a hyperimmune state may continue even after HSV-1 replication has stopped (218). This theory supports the potential role of immunomodulatory therapies to promote HSE recovery (218).

One explanation for why HSV-1 specific IgG may persist in CSF, with HSE still developing despite their presence is that HSV-1 may evade the effector functions of antibodies such as phagocytosis and complement lysis (219). HSV-1 has been found *in vivo* to encode glycoproteins which are capable of interacting with complement (gC) and binding to the Fc region of IgG (gE) (219). As a result the activity of complement components C1q, C3, C5 and properdin and cellular toxicity normally co-ordinated by antibody is blocked (219). Further supporting this, elimination of C3 and IgG Fc binding which is usually co-ordinated by gC and gE, respectively increased the efficacy of HSV-1 antibody and complement neutralization using mice infected intraperitoneally with HSV-1 (219). *In vivo* severity of infection measured by viral titre and mortality also decreased indicating that HSV-1 mediated evasion of IgG may increase susceptibility to HSE (219). The fact that the persistence of HSV-1 specific IgG is insufficient to prevent development of HSE indicates that a synchronous innate and cell-mediated immune response may facilitate the control of HSV-1 in the CNS (21).

Germinal centre (GC) reactions allow for B cells to refine their antigenic binding capacity via somatic hypermutation, upregulation of chemokine expression and class-switch recombination (21, 220). GC reactions could underlie the B cell response associated with HSV-1, as studies using mice infected intra-ocularly with HSV-1 demonstrated elevated HSV-1 CD19+CD27+ memory B cells alongside class switched IgG+CD19+CD27+ memory B cells in rodents which were asymptomatic (221). However, GC reactions could be pathogenic, as they may contribute to the B cell response underlying the development of post-HSE AE (21). Post-HSE AE is generally defined clinically as sustained (> 24 hour) worsening of neurological function despite no HSV-1 DNA being present in the CSF (222). Evidence finding IgA, IgM and class-switched IgG antibodies specific to NMDAR within the CSF of 13 patients (223) alongside elevated chemokines including CXCL13 and CCL19 in one patient (224) with post-HSE AE supports that these GC reactions could contribute to onset of pathology, however studies using larger samples are required (21). Emerging data suggests cervical lymph nodes and meningeal lymphatics mediate an overlap between the brain and periphery, indicating a potential mechanism by which GC reactions could promote complications within the CNS (21, 225, 226). Relapses in HSE cohorts have been predominantly in the presence of NMDAR autoantibodies, with these patients responding well to steroid therapy (60, 61, 227). Other autoantibodies which have been associated with post-HSE AE include those which target CASPR2, GABA-A and the dopamine 2 receptor (222).

### 3.8 Neuronal injury

CNS damage post HSV-1 infection occurs due to lytic infection of neurons and glial cells and the induction of neuroinflammation (228). Viral-induced neural and glial cell death may arise in acute HSE, as observed using human HSE autopsy and surgical biopsy samples taken from 10 patients (229). HSV-1 induced apoptosis has been reported to occur via the intrinsic pathway, with the translocation of Bax into the mitochondria triggering cytochrome c release and caspase 9 activation in human epithelial Hep-2 cell cultures (230). Induction of apoptosis in neurons may contribute to alterations in neurotransmitter and growth factor secretion (229).

Infection of isolated cortical neuron cultures has also been demonstrated to reduce dendritic spine and secondary dendrite numbers (231). This could be explained by the concomitant increase in Arc protein expression observed in these cultures, which inhibited the synthesis of post-synaptic density scaffolding proteins such as PSD-95, Drebin and CaMKIIβ which typically maintain the spinal architecture (231). Alongside this, HSV-1 infected neurons exhibited no response to glutamate stimulation indicating dysregulated synaptic transmission (231). Alterations in long-term potentiation within the hippocampus have been attributed to elevated levels of IL-1β, which led to increased expression of MeCP2 and synaptic deficits in mice intra-nasally infected with HSV-1 (232).

## 4 Factors impairing cell-mediated immunity to HSV-1

The cell mediated immune system therefore has the capacity to act as a ‘double-edged sword’ which can serve to both protect the host against HSE development however also increase vulnerability to the disease if the immune response is dysregulated (13). Further factors may pre-dispose individuals to developing HSV-1 encephalitis including viral evasion strategies (viral proteins, inhibition of the unfolded protein response) (164, 233–237), external factors (stress, immunosuppressive treatments) (179, 238–240) and host genetic pre-disposition (TLR3 deficiency, Rel mutations) (241–243). Exploration of each of these factors reveals an underlying theme: impaired activation and/or effector function of T cells (244).

### 4.1 Viral immune evasion strategies

The antiviral action of innate immune mediators such as type I and type III IFNs may provide HSV-1 protection in peripheral tissues, however HSV-1 can eventually invade endings of sensory nerves and undergo retrograde axonal transport to reach the ganglia of peripheral nerves (245, 246). HSV-1 can either continue replication resulting in neuronal death or establish latency, with the mechanisms underlying why one may arise over the other still unclear (110, 236, 245, 247, 248). The specific aetiologies of HSV-1 reactivation following latency and the variable outcomes following this are also unclear, however evidence supports that reactivation is more frequent in the immunocompromised (40, 236, 249). Importantly, there is also evidence suggesting HSV-1 can employ strategies to evade innate and adaptive immune responses with some examples of these listed below (236, 250–253).

#### 4.1.1 Viral proteins

ICP0, an immediate early protein (IE), can inhibit the innate immune response to HSV-1 by targeting MyD88 and Mal for proteasomal degradation and therefore preventing IFN production and the ability for a downstream immune response to arise (233–236, 254). Alongside this, ICP0 drives the movement of USP7 from the nucleus into the cytoplasm, where USP7 prevents TLR-dependent NF- κB and JNK signalling through the deubiquination of TRAF6 and IKKγ (255). VP16 is a further tegument protein which also inhibits NF- κB signalling and type I IFN expression by targeting the p65 subunit (256). VP22 also prevents type I IFN synthesis, this time by interacting with cyclic guanosine monophosphate-adenosine monophosphate synthase (40). UL36 is able to target and inhibit IFN- β production by deubiquitinating TRAF3 resulting in impaired activation of TBK1 and prevention of IRF3 dimerization meaning the immune response can be evaded (236, 257).

HSV-1 proteins enable evasion of the cell-mediated immune response (164, 258–262). Viral peptides, as a result of presentation via MHC-I molecules, are ordinarily recognised by CD8+ T cells and subsequently eliminated (258, 263). As a result, a virus which alters the MHC-I pathway could be protected against CD8+ T cells (259). HSV-1 encodes the viral proteins ICP47 and US3 which in cell culture downregulates MHC-I expression and as a consequence inhibits antigen presentation via MHC-I and prevents CD8+ T cells from carrying out their typical effector functions (165, 260, 261, 264). A further protein encoded by HSV-1, UL13 kinase, prevents CD8+ T cell accumulation through downregulation of CXCL9 and CXCL10, preventing CD8+ T cells from mounting a sufficient immune response in the CNS (259), most likely owing to reduced recruitment of CD8+ T cells to infection sites resulting in a diminished immune response (259). In support of this, delayed infiltration of CD8+ T cells specific to HSV-1 was shown to promote HSE (262). Therefore, HSV-1 may exert its pathological effects through downregulation of CXCL9 and CXCL10 at infection sites (259). Supporting this, directly injecting CXCL9 and CXCL10 into the CNS of mice with HSE led to an increase in CD8+ T cells located in the CNS and reduction in mortality (259). This suggests that the viral proteins ICP47, US3 and UL13 kinase, in concert, may downregulate the CD8+ T cell response which would usually confer protection against development of HSE (259).

#### 4.1.2 Inhibition of the unfolded protein response factor inositol-requiring enzyme 1 alpha

A further mechanism by which HSV-1 subverts the hosts immune system is through infection of dendritic cells (DCs), impairing their normal function, reducing their capacity to activate T cells, and resulting in their apoptosis (237). Inoculation of DCs *in vitro* resulted in a 50 fold increase in splicing of the mRNA of XBP1 (265), which could therefore activate Inositol-Requiring Enzyme 1 alpha (IRE-1α), a key mediator of the unfolded protein response (UPR) (266). The UPR is usually initiated when misfolded proteins accumulate within the endoplasmic reticulum (ER) (267), but it can also be triggered by viral infection (268) or the release of inflammatory factors (269). HSV-1 infection can suppress the IRE-1α UPR pathway, resulting in reduced synthesis of viral proteins (270) and promotion of DC apoptosis which together result in poor activation of cell-mediated immunity (271). In contrast, experimental upregulation of IRE-1α following HSV-1 infection of DCs isolated from mice and then injected into the footpads 6 hours later promoted DC migration to lymph nodes and subsequent activation of HSV specific CD4+ and CD8+ T cells (237, 272). These findings suggest that IRE-1α may function as a central coordinator of adaptive immune responses, highlighting its potential as a future therapeutic target (237).

### 4.2 External factors

#### 4.2.1 Stress

Psychological stress in the murine model appears to increase susceptibility to HSV-1 infection and its progression to HSE (180). In mice stress suppresses MHC-I expression and reduces CD8+ T cell numbers in cervical lymph nodes through altered glucocorticoid receptor (GR) activity (180), possibly by inducing apoptosis of CD8+ T cells (273) by downregulation of the anti-apoptotic protein bcl-2 (274). Similar reductions in CD8+ T cells have been observed in popliteal lymph nodes of mice following footpad infection (275). Stress has been shown *in vivo* to reduce microglial H-2Kb expression impairing presentation of the dominant HSV-1 epitope gB498-505 to CD8+ cells in the CNS (276). In addition, stress reduces IL-2 synthesis, limiting clonal expansion of T cells (277). Collectively, these mechanisms may contribute to reduced CD8+ T cell numbers in the CNS, potentially reducing the protective cytotoxic and neuroprotective effects that these cells may provide in prevention of HSE (178, 179).

#### 4.2.2 Immunosuppressive treatments

Case reports highlight patients receiving azathioprine for inflammatory bowel diseases who have developed HSE; including a fatal case with cerebral herniation (240, 278). Azathioprine induces T cell apoptosis (32), possibly reducing protective CD8+ T cell infiltration of the CNS (279).

Alemtuzumab, by depleting T cells, has been linked to viral encephalitis in allogenic stem cell transplant recipients (280). Several case reports and a retrospective study of cancer patients (281–290) demonstrate that the use of immunosuppressive treatment with brain radiation and/or corticosteroid therapy can increase HSE predisposition. In this case, administration of solely whole-brain radiation therapy or high-dose dexamethasone was found to induce HSE (289, 291, 292). Finally, long-term treatment with corticosteroids has been shown to reduce CD4+ T cell counts, limiting the efficacy of the cell-mediated immune response and consequently increasing the risk that infections will develop following administration of radiation therapy (293).

Natalizumab, a monoclonal antibody which targets α-4 integrin, is used to manage multiple sclerosis and Crohn’s disease (32). As α-4 integrin is a cell adhesion mediator which inhibits the relocation of peripheral cells into the CNS, use of natalizumab can lead to a lower CSF CD4+/CD8+ T cell ratio than was present in the blood, resulting in impaired immune surveillance and an insufficient cell-mediated immune response (294). One case series followed 20 patients with multiple sclerosis treated with natalizumab who later had confirmed herpes virus infections, of which half the cases presented with HSE (295).

### 4.3 Inborn errors of immunity

Cell-mediated immunity has not yet been found to confer increased protection against childhood HSE, as children with severe combined immunodeficiency (SCID) (which impairs T and B cell responses) do not have increased predisposition to HSE (296). However, deficiencies in NK cell function may be a contributing factor (297, 298). Children with TLR3 mutations are predisposed to develop HSE, are at risk of relapsing disease and often require prolonged antiviral prophylaxis (241). Mutations of genes involved in the downstream TLR3 pathway have also been found in children who have HSE, including *UNC93B1, TRIF, TRAF3 and TBK1* (49). However, it is important to consider that only a small fraction of children (5%) with HSE have been previously found to carry mutations in TLR3 (241). The small sample sizes indicate that large-scale population studies determining the exact prevalence are required to understand the clinical relevance of TLR3 deficiency (299). Two cases have recently been reported in adults with mutations impacting expression of mannan-binding lectin serine protease-2 (MASP-2), which is key in initiating the lectin pathway of the complement cascade (300). The lectin pathway co-ordinates the recognition of carbohydrate and mannan structures by PRRs, allowing MASP-2 to bind and initiate C2 and C4 cleavage to facilitate downstream opsonisation and lysis of infected cells (32). This mutation has been implicated in inhibiting this pathway via either preventing activation of MASP-2 or preventing protein secretion (300). Recently, two separately reported patients who had childhood HSE had homozygous mutations for rare deletions in the *TMEFF1* gene which typically facilitates expression of a IFN-I independent neuron restriction factor which could prevent CNS dissemination (301). *TMEFF1* has been shown *in vivo* to interact with NECTIN-1, which is an extracellular domain which acts as a receptor for HSV-1 permitting fusion and entry of the virus (301). As a result, *TMEFF1* deficiency could enhance gD-NECTIN-1 mediated fusion of the virus into the cell (301). A recent genome wide analysis using whole exome sequencing from patients with forebrain HSE found five unrelated patients to have rare monoallelic mutations in the *SNORA31* gene (302). Deletions in *SNORA31* have been found *in vivo* to increase susceptibility of hPSC-derived cortical neurons to HSV-1 infection (303). Transcriptome analyses of these neurons revealed that this mutation impaired overarching immune responses however TLR3 and IFN-α/β signalling remained intact (303). This suggests the presence of a further, undiscovered mechanism underlying inborn errors of immunity to HSV-1 which pre-dispose individuals to HSV-1 which requires further investigation (46, 304).

TLRs are a sub-type of PRR which are able to detect unique pathogen structures and initiating inflammatory processes such as cytokine expression, phagocytosis and autophagy to link innate and adaptive immunity (305–307). Two children with an autosomal recessive mutation in the UNC-93B which co-ordinates TLR3, TLR7, TLR8 and TLR9 signalling were observed to develop HSE (304, 308). This same study observed that peripheral blood mononuclear cells (PBMCs) demonstrated reduced IFN-α, IFN-β and IFN-λ production attributed to TLR3 deficiency alone, indicating clinical implications for TLR3 which may not be applicable to other TLRs (304). TLR3 also initiates an immune response following detection of viral double-stranded RNA (dsRNA), which is significant as dsRNA is an essential form of viral DNA found in HSV-1 (136, 309). Mutations in TRIF, a TLR3 adaptor protein, and the transcription factor IRF3 have been reported in patients (both adults and children) with HSE (45, 49, 310). Autosomal dominant mutations in TRAF3, which may play a role in type I and III IFN production has also been noted in a child with HSE (47). Other mutations, including those in NF-κB essential modulator, tyrosine kinase 2 and STAT1 have the capacity to alter IFN and cytokine production therefore are implicated in susceptibility to a vast array of infections, with HSE being a notable example (310–312).

In fibroblasts, TLR3 activation induces IFN-β and -λ (313), while in dendritic cells it supports cross-presentation of viral antigens (314), facilitating antiviral T cell responses (315).Supporting this, TLR3-deficient mice show markedly reduced HSV-specific CD8^+^ T-cell numbers and IFN-λ production after cutaneous infection (316). Loss of IFN-λ signalling is significant as it could compromise neuroprotection, apoptosis, and MHC-I upregulation, diminishing antigen presentation to CD8^+^ T cells (21, 317). Pre-clinical work using mice with corneal HSV-1 infection found that STING-IFN-λ signalling can inhibit HSV-1 dissemination to the CNS, which could explain for why reduced IFN-λ production could confer increased HSE susceptibility (318). Importantly, there is incomplete penetrance of the TLR3 signalling pathway, as relatives with the same mutation do not always develop HSE following infection with HSV-1 (32). Alongside this, TLR3 signalling abnormalities have not been reported to confer an increased risk to other infections (32). These children do not have an increased susceptibility to peripheral HSV-1 infection, suggesting that TLR3 may not provide host defence outside of the CNS (46, 313). It has been suggested that HSE predisposition in the CNS is due to type I IFN production following TLR3 stimulation by nonhematopoietic cells which have been infected with HSV-1 (32). Supporting this, induced pluripotent stem cells from patients with deficiencies in TLR3 and UNC93B which differentiated into neurons and oligodendrocytes had increased susceptibility to HSV-1 infection compared to controls (319).

Therapeutic modulation of TLR3 signalling shows promise. Poly(I:C) (a TLR3 agonist) stimulation before HSV-1 challenge reduced brain viral load and mortality in mice (46), whereas, possibly due to increased expression of IFN-β and CCL5, antagonism of TLR3 during intracerebral infection unexpectedly enhanced survival rates (314).

### 4.4 Mutations found in murine models

#### 4.4.1 Ptprc L3X

Mutations in the Ptprc gene have not to date been reported in patients with HSE limiting the utility of this research overall (320). Caignard et al. propose that the Ptprc gene may still be functionally important in generating T cell responses in humans despite no mutations in the gene being reported to date (320). Mice derived from the C57BL/6 genetic background were treated with N-ethyl-N-nitrosourea to artificially induce the Ptprc L3X point mutation which disrupts thymocyte development and results in a T cell deficiency (320). Increased HSV-1 viral titres in 2 out of 5 of the mice and up-regulation of mediators which induce BBB damage such as MCP1, IL-6, MMP-3 and MMP-8 was observed, indicating genetically induced T cell deficiency may increase HSV-1 susceptibility in mice (320–328).

#### 4.4.2 RelC307X

A truncation mutation in the c-Rel transcription factor (RelC307X) has been implicated as a driver of HSE by research using mice intranasally infected with HSV-1 (243). In these mice, the RelC307X mutation led to increased rates of viral replication, upregulation of IFN-dependent genes (243), and increased IFN expression- processes linked to neurotoxicity and tissue injury (329, 330). Elevated expression of inflammatory mediators including interleukin 1 receptor agonists, CCL2 and CXCL10 correlated with HSE development (243). Infiltration of T and myeloid cells into the CNS was delayed (243),, possibly driven by CCL2 and CXCL10-mediated recruitment of CXCR3+ T cells and CCR2+/CXCR3+ monocytes into the brainstem (331–333), resulting in a dysregulated cell-mediated immune response and more severe pathology (243). Survival outcomes reflected this immune dysfunction: 60% of mice with the RelC307X mutation developed HSE and succumbed within 6 to 9 days; whereas no wild-type mice developed CNS disease (243, 334).

Together, these findings suggest that c-Rel deficiency disrupts the cytokine environment and immune cell recruitment, predisposing to uncontrolled CNS inflammation (243, 331–333). The *REL* gene in humans has been associated with other forms of inflammatory disease (335, 336), with a recent case report finding a c-Rel deficient patient to be more susceptible to human herpesvirus-5, *Cryptosporidium* and *Salmonella* (242). Observations of c-Rel deficiency in humans is currently limited to a very limited number of case reports involving individual patients limiting its clinical application to HSE overall (242). Additionally, the RelC307X mouse remains a purely experimental model which demonstrates the role of pathological T cell infiltration in the unregulated neuroinflammation and viral replication seen in HSE until the mutation is identified in major population databases (243).

#### 4.4.3 Lymphotoxin α

Equally, while lymphotoxin α (LTα) deficiency owing to genetic polymorphism has been explicitly reported in mice, it has rarely been reported in humans with the exact prevalence currently unknown (337). However, LTα knockout mice infected with both systemic and cutaneous HSV-1 exhibited increased susceptibility to HSE compared to B6 mice, developing encephalitis more rapidly and following exposure to lower viral doses (338). B6 mice were used as controls due to their capacity to exhibit a Th1-biased response (339) The increased susceptibility could be explained by the observation LTα-/- mice generated a ~10 fold weaker CD8+ T cell response alongside a 2-fold reduction in IFN-γ secretion compared to B6 mouse controls (338). A diminished IFN-γ response may increase susceptibility to HSE through a variety of mechanisms including impaired antigen presentation (340) and reduced cytotoxic activity mediated by bcl-2 expression (341). Additionally, the spleens of LTα−/− mice were deficient in chemokines such as CCR7 (338, 342). Chemokines such as these are important in facilitating activation of mature dendritic cells and naive T cells ultimately allowing CD8+ T cells to mount an immune response to facilitate protection against HSE (343–345).

Administration of LTα−/− mice with plasmids that promoted expression of CCR7 ligands resulted in CD8+ T cells activation and effector function was re-established, demonstrated by improved cytotoxic action of the cells and increased IFN-γ production (346). CD8+ T cell responses to HSV infection could be further enhanced with additional administration of plasmid DNA encoding CCL19 or CCL21, indicating that chemokine immunotherapy can compensate for immune defects in LTα−/− mice and could be a future avenue for immunotherapy in humans (346).

The research on lymphotoxin-α deficiency is limited by current observations being restricted to mouse models and often with conflicting results (337). For example, its contribution to host defence has been challenged by the fact that LTα knockout mice were still able to produce sufficient TNF to mount an immune response (337). Additionally, the mechanisms that LTα uses to promote inflammatory processes are still poorly understood indicating the need for research distinguishing between their currently overlapping immunological functions (337).

## 5 Cell-mediated immunity and cytokines

Cytokines are key to the activation of cell-mediated immunity and promoting the migration of leucocytes into the CNS, therefore serving as a bridge between innate and adaptive immunity (347). Understanding the cytokines that correlate with better and worse outcomes respectively in patients with HSE, including the ones outlined below, can be used to explore future potential immunomodulatory treatments (348).

### 5.1 IL-1 superfamily

IL-1α and IL-1β are both implicated in HSE pathogenesis, and are both offset by IL-1RA via the IL-1 receptor 1 (349, 350). Preliminary IL-1 induction drives expression of proinflammatory chemokines and adhesion molecules, increasing BBB permeability and predisposition to encephalitis (349–351). For example, astrocytic IL-1α promotes CXCL1 release, enabling neutrophil transmigration and BBB disruption (112), while microglial IL-1 β is upregulated following HSV-1 infection (125, 350–352). IL-1β may enhance the differentiation of Th1 and Th17 cells in autoimmune encephalomyelitis, contributing to the microglial activation, neuronal damage and neuroinflammation which was then observed (353). In terms of HSE, experimental models link elevated IL-1 to clinical features characteristic of HSE including fever, increased BBB permeability and cerebral oedema (125, 354–356).

There is evidence to suggest that inflammasomes, which are mechanisms of inflammatory signalling primarily mediated by microglia during infection, may contribute to the activation of pro-inflammatory cytokines IL-1 β and IL-18 during HSE (357). Mice who were deficient in an inflammasome adaptor protein, had significantly lower titres of IL-1β and IL-18 in the brain and significantly prolonged survival (357). This suggests that the pathogenic increase in pro-inflammatory cytokines seen in HSE could be co-ordinated by glial cell mediated inflammasome activation indicating an additional role of cell lines of the adaptive immune system may have following the initial innate immune response (357).

Clinically, Michael et al. (358) found serum IL-1RA was the mediator most significantly associated with a good outcome in infectious encephalitis patients, whereas a high IL-1beta:IL-1RA ratio predicted poor outcome. This is likely to reflect rapid, localised IL-1 production in the CNS, with lower peripheral titres (358).

Together, these findings highlight the balance between IL-1 and its antagonists as a key determinant of BBB integrity, disease severity and outcome (357, 358).

### 5.2 IL-6

Interleukin-6 (IL-6) may act as an additional mediator of the cell-mediated response, and is secreted following activation of the NF-κB and TLRs post initial HSV-1 infection (359). IL-6 then has the capacity to support T cell survival and differentiation and other cells in the CNS to stimulate release of further cytokines/chemokines including TNF and IL-1β potentially a factor mediating an excessive inflammatory response (359).

### 5.3 IL-10

*In vitro* studies show that HSV-infected human microglia produce interleukin-10 (IL-10) which, together with lymphocyte-derived IL-10, suppresses proinflammatory cytokines such as IL-1 and promotes cell-survival signalling (360, 361). IL-10 additionally reduces cyclooxygenase-2 production; a key regulator of BBB permeability. In murine models it correlates with reduced neuronal death (361, 362). Clinically, higher IL-10 levels are associated with favourable outcome indicating its feasibility as a biomarker indicative of a better prognosis (358). Additionally, a strong negative correlation has been found between IL-10 levels and the CSF:albumin ratio in patients with encephalitis, indicating higher IL-10 levels are associated with a reduction in BBB permeability (358). These findings suggest that IL-10 protects against excessive neuroinflammation by stabilising the BBB and limiting proinflammatory mediators (358). Evidence from Japanese encephalitis virus infection supports the link between IL-10, GCS and BBB permeability (363).

IL-10 secretion is a characteristic feature of Treg cells, which are a subset of CD4+ T cells distinguished by their Foxp3+ expression and ability to inhibit pro-inflammatory pathways (207, 209, 364). Infection of fetal microglial cells with HSV-1 found that IL-10 selectively reduced synthesis of TNF, CCL5 and IL-1β, indicating IL-10 may inhibit production of cytokines and chemokines ordinarily produced following HSV-1 infection (361). However, there is conflicting research debating which class of CD4+ T cells mediate IL-10 induced neuroprotection (365). For example, when mice were pre-treated with either polysaccharide A (PSA) or a placebo (PBS) and infected with HSV-1 via corneal scarification, depletion of Tregs did not impair neuroprotection (365). Instead, CD39+CD73+ T cells alone conferred sufficient HSE protection in mice (365). As a result, the precise role of Tregs in IL-10 production is still unclear and more studies directly assessing the role of Treg mediated IL-10 secretion are required.

### 5.4 IFN-γ

The neutralisation of IFN-γ abrogates T-cell-mediated recovery following acute HSV-1 infection, supporting its central role in antiviral immunity (366). Produced by both CD4^+^ and CD8^+^ T cells, IFN-γ promotes viral clearance, as demonstrated by impaired resolution of HSV-1 in footpad tissue after antibody blockade (367). Mechanistically, IFN-γ induces an antiviral state via activation of the 2,5-oligo(A) synthetase pathway and phosphorylation of the α subunit of eIF-2, inhibiting viral protein synthesis (340). It also activates macrophages (368) and upregulate expression of class I and II MHC (340) Supporting this, neutralisation of IFN-γ in mice following cutaneous infection with HSV-1 were less able to clear the infection and generate a sufficient T cell response (366).

Mice deficient in IFN-γ also have increased susceptibility to HSE (369, 370), partly due to loss of neuronal protection mediated by IFN-γ-induced bcl-2 expression (341). Since lower HSV-1 specific CD8+ T cell numbers correlate with increased disease severity and HSV-1 viral load in intra-nasally infected mice (262), impaired IFN-γ production may limit cytotoxic activity against infected cells (180).

### 5.5 IFN-α

In early HSV-1 infection, IFN-α acts as an immunomodulator and co-ordinates the expression of viral immediate-early gene transcription (371). IFN-α subsequently supports the adaptive immune system by enhancing cytotoxic T lymphocyte activation, upregulation of antigen presentation via MHC-I and MHC-II and promotes secretion of additional cytokines (371).

### 5.6 TNF

TNF facilitates microglial activation and regulates NK and lymphoid cell differentiation (372, 373). In murine HSE, TNF-α transcripts are detected within microglia, CNS infiltrating monocytes and macrophages (119).TNF also has the capacity to induce and regulate both the innate and adaptive immune responses (32). This has been demonstrated in murine models as TNF deficiency enhanced viral replication and suppression of the typical innate immune response, resulting in increased HSE susceptibility compared to wild-type counterparts (119, 374, 375). Clinically, HSE has occurred in patients who were treated with anti-TNF therapy, consistent with loss of TNF-dependent immunity (376).

## 6 Potential therapeutic avenues: modulating cell-mediated immunity

Understanding the molecular mechanisms underlying the cell mediated immune response to HSE provides new insights for the use of different immunomodulatory strategies including corticosteroids (377), anakinra (378), tocilizumab (379, 380) and targeted vaccine strategies (381). However, optimal management in terms of duration of therapy, use of adjunctive immunomodulatory strategies and how to respond to therapeutic failure are still uncertain therefore further prospective trials using human participants are required to inform evidence-based treatment algorithms which can improve outcomes for patients (382–384).

### 6.1 Corticosteroids

Several studies to date have evaluated the use of corticosteroids as an adjunctive therapy for patients with HSE (382, 385–390), including case reports and series which report that corticosteroids alone reduced cerebral oedema and intracranial pressure in both children (391, 392) and adults (393–395). Their use remains controversial, as despite their potential to reduce cerebral oedema and suppress the immune response there is concern that the resultant immunosuppression may facilitate uncontrolled viral replication (390, 396). For example, a case series involving six children with HSE found that despite the three patients in receipt of adjunctive steroid therapy exhibiting improved motor function, cognition and seizure control, abnormal radiological findings were similar across both groups (397). This indicates that corticosteroid therapy could improve symptom control however underlying neurological damage from viral replication may persist (397). Furthermore, production of CXCL1 was found to continue following acute administration of dexamethasone following intra-cranial HSV-1 infection of mice indicating that therapies which are more specific may be required (112, 390).

Mechanistically, corticosteroids suppress cell-mediated immunity (398), and downregulate pro-inflammatory cytokines including IL-6, IL-1β and TNF, which may impair control of the virus (398). Findings from a non-randomised retrospective study found that administration of either dexamethasone or prednisolone (22 patients) as an adjunctive therapy demonstrated improved neurological outcomes compared to patients who only received aciclovir (23 patients) (387). This improved outcome may have been due to an overall pro-inflammatory cytokine reduction, with levels of cytokines such as IL-6 (128). However, in a study involving intraperitoneal administration of aciclovir and cortisone one day after intranasally infecting mice with HSV-1, no significant difference in viral load was observed indicating that immunomodulation with this drug didn’t lead to any additional benefits compared to aciclovir alone (32, 399). The variations in pre-clinical and clinical outcomes could be explained by fundamental differences between experimental mouse models. Unlike in humans, HSV-1 is unable to spontaneously re-activate in mice and co-infection is less likely to arise (389). Results from a randomised trial (DexEnceph NCT03084783) are expected which will inform use of steroids in HSE which can incorporate the heterogeneity of human outcomes which are seen in clinical practice (377).

There is conflicting evidence regarding whether adjunctive therapies such as glucocorticoids and intravenous immunoglobulin (IVIG) may be beneficial, as despite evidence in mice for IVIGs (400–402) some case reports where patients receive both anti-viral, steroid and IVIG treatment report clinical improvement (390, 394, 397, 403, 404) whereas others report either a variable (387, 405) or poor (406) prognosis overall. No randomised controlled trial to date has confirmed the efficacy of adjunctive treatments so far and therefore are essential (387, 390, 407, 408). Alongside this, optimal timing of immunomodulatory treatment administration is also uncertain. Prompt administration of steroids is usually advocated for the management of CNS infections including bacterial meningitis, however delayed glucocorticoid administration (3 days after infection) in mice intranasally infected with HSV-1 exhibited reduced cytokine production, viral load and reduced mortality (409). Animal models indicate that HSV-1 replication rises exponentially within the first 4 days of inoculation, with a predominant CNS pro-inflammatory response involving expression of NOS, matrix metalloproteinases, cytokines (IL-1, IL-6, TNF) and chemokines (CXCL9, CXCL10) between days 4-8 (10, 399, 409–413). Theoretically, timing the administration of corticosteroids to coincide with the inflammatory response that arises at a later stage of disease could prevent the cerebral disease which may arise due to excess inflammation (13). Equally, there is evidence suggesting that initiation of steroid therapy too early may also lead to harmful consequences (389). One mechanism HSV-1 may use to invade neurons includes inhibition of NOS therefore early steroid administration (which inhibit NOS) may promote viral dissemination to the CNS (399, 414). Supporting this, induced NOS inhibition in animals prior to HSV-1 infection led to increased mortality and viral titres compared to when treatment was started 1-3 days post infection (131, 399, 415).

Repeated HSV-1 infection of the corneal mucosal surface may result in HSK and prophylactic topical corticosteroids may be used alongside oral anti-viral therapies to prevent this from arising (104, 416, 417). Drugs which are currently used as anti-inflammatories in HSK include prednisolone, dexamethasone and cyclosporine (418–421). However, although corticosteroids may reduce pain and lower inflammation, a previous review conducted suggested that corticosteroid treatment may activate glucocorticoid receptor (GR) activation which may initiate HSV-1 re-activation and suppression of the typical immune response to HSV-1 (422). Additionally, prolonged prophylactic steroid use may lead to adverse effects including glaucoma and cornea thinning meaning the overall evidence supporting their use for HSK is conflicting (423–425).

It is imperative that the use of corticosteroids as an adjunctive treatment is balanced with the potential for adverse effects (398). Corticosteroids are able to suppress the cell-mediated response by impairing NK and CD8+ T-cell function which may diminish capacity to counteract viral replication and increase susceptibility to secondary infections (398, 426–428). There is limited clinical data based on patients with HSE, however a retrospective cohort study of patients hospitalised with West Nile virus, corticosteroid-induced immunosuppression was associated with increased mortality, potentially due to enhanced viral replication during acute infection (429). Furthermore, there have been examples of case reports where high-dose steroid treatment has been associated with development of atypical HSE suggesting that corticosteroid administration may alter gene expression to facilitate a transition from latent to lytic replication (388, 430). This further supports the notion that implementation of corticosteroid treatment at a later stage after antiviral therapy may facilitate more effective viral clearance (389). As a result, clear evaluation of whether the benefits outweigh the risks is essential in the consideration of adjunctive corticosteroid therapy (32, 398).

### 6.2 Anakinra: IL1 receptor antagonist

Anakinra is a recombinant IL-1RA administered subcutaneously, and has previously received approval for inflammatory disease management of conditions such as rheumatoid arthritis (RA) and Still’s disease (431). Antagonising IL-1β in patients with HSE could keep the cell-mediated immune response controlled through inhibiting the activation of excess microglia and cytokines such as TNF, IL-6 and IFN-γ which would ordinarily promote recruitment and infiltration of T cells into the CNS (119). As a result, anakinra could inhibit excess infiltration of CD4+ and CD8+ T cells, ensuring the adaptive immune response remains neuroprotective rather than a source of the neuronal damage and excessive inflammation which may be seen in patients with HSE (432). The benefits of IL-1RA have also been reported in a case reports involving patients with microglia-dominant brain neuroinflammation (433) and chronic autoimmune meningoencephalitis (378). Alongside this, HSV-1 induced IL-1β may upregulate the mediator MeCP2, which increased expression of genes c-fos and syn1 while decreasing expression of protein resulting in cognitive decline and synaptic dysfunction (232). In mice infected with HSV-1, administration of anakinra was unable to restore memory in early stages of HSV-1 replication, however beneficial effects could be seen in subsequent weeks (232). This suggests that changes in memory and cognitive function induced by HSV-1 replication extend far beyond the action of IL-1β, and therefore administration of anakinra may be more beneficial in later stages of the disease when viral replication has been controlled (232).

As higher levels of IL-1RA were significantly associated with better outcomes, early antagonism of IL-1 could be a viable therapeutic target (358). Furthermore, it is important to assess the critical time frames where therapeutic interventions are most efficacious, as has already been demonstrated by studies looking at the use of glucocorticoid therapies and murine models (374, 434). In HSV-1 infected mice, Targeting the protective effects of IL-1β has potential, supported by the fact that IL-1β knockout mice had increased mortality following intranasal HSV-1 infection (374).

### 6.3 Tocilizumab: IL-6 receptor blocker

Tocilizumab is a humanised IgG1 monoclonal antibody and a potent anti-inflammatory agent which binds to both membrane-bound and soluble IL-6 receptor, preventing its downstream signalling via JAK/STAT3, MAPK and PI3K pathways (435). This has pleiotropic effects on innate and adaptive immune responses (436). It has previously been used in small numbers of cases for patients with refractory autoimmune encephalitis (AE) who are resistant to the first- or second-line immunosuppressive therapies (379, 380). Excessive IL-6 production contributes to the dysregulation of cell-mediated immunity through stimulating excessive differentiation of CD8+ cytotoxic T cells, resulting in additional excitotoxicity-induced neuronal damage (437). Therefore, by binding to the IL-6 receptors, tocilizumab could be a viable way of reducing neuronal damage and lowering inflammation overall (435). Administration of anti-IL-6 antibodies shortly before thermal stress induced re-activation has been found to reduce frequency of viral re-activation in mice with corneal HSV-1 infection, suggesting that early administration could prevent viral dissemination and prevention of progression to encephalitis (438). However, IL-6 has been demonstrated *in vivo* to also exert a protective effect by protecting neural progenitor cells and neuroblasts via STAT3 signalling in cell cultures infected with HSV-1 (439). This suggests that blockage of IL-6 too early into disease could also be harmful, therefore further research using encephalitis models is required to understand optimal timing of administration (439).

### 6.4 Immunisation

The main HSV-1 vaccine rationales which have been proposed include prophylactic (subunit vaccines/replication-defective/mRNA-based vaccines which can induce neutralising antibodies and/or immunological memory) (440–442), therapeutic (DNA/mRNA/vector-based vaccines which express antigen to stimulate a cell mediated immune response) (443–445) or combined (multi-antigen DNA/mRNA/nanoparticle adjuvant vaccines which target cellular and humoral immunity simultaneously) (446–448) approaches. To date, despite prophylactic HSV-1 vaccines showing initial potential in animals, the replication in human trials has been lacking (449). For example, a subunit vaccine developed by Agenus did not have progression past phase 1 of development (449). Currently, other candidates are undergoing clinical trials such as mRNA-1608 (Moderna’s mRNA vaccine) and 0ΔNLS (Rational vaccines’ live-attenuated vaccine) (449). However, as CD8+ T cells may be protective against future development of HSE, a vaccine which targets CD8+ T cells may be effective in preventing lethality associated with the disease overall (381). Other approaches hypothesised for HSV-1 vaccine design include attracting Trm cells in peripheral tissue locations (29). Immunisation with d301 induced formation of HSV-1 CD4+ and CD8+ T cells which, mediated by IFN-γ, were able to reduce replication of HSV-1 and could therefore be used as a prophylaxis against HSE (450). Understanding the genetic pre-dispositions which mean that HSE develops in some HSV-1 infected individuals but not others, such as the Ptprc L3X and RelC307X mutations can be used to determine who would most benefit from receipt of a vaccine (242, 320). Furthermore, the creation of an experimental model of the RelC307X mouse means there is scope for the gene itself or further downstream inflammatory mediators to be targeted, preventing the aberrant immune response and long-term sequelae associated with HSE (243).

### 6.5 Reasons for therapeutic failure

Significantly poorer outcomes are associated with a delay in aciclovir initiation, with longer delays increasing the severity of downstream neurological sequelae (434, 451–454). Clinical factors contributing to this include a lack of clinical suspicion for HSE while waiting for results of CSF PCR and brain imaging not being immediately conducted following admission, indicating the importance of commencing aciclovir while awaiting CSF analysis results (452, 454, 455). Treatment failure may also arise due to relapse, with one study reporting this to arise in 12% of patients treated with aciclovir (456). This may be due to autoimmunity, underlying immunosuppression or immune defects however the precise reasons underlying this are still unclear (14, 457).

Viral resistance to aciclovir in immunocompetent individuals is rare, arising in < 1% of cases (458). However, immunocompromised individuals appear to be at a higher risk, with up to 30% of those who have had a bone marrow transplant being reported to be resistant (459). As a result, if a patient is deteriorating or not responded in the presumed way to management then resistance should be considered as an underlying cause (459). Aciclovir resistance in patients with HSK is up to 6.4%, with predisposition increasing in patients who have been taking aciclovir prophylactically for over a year (460–466). Previously described mechanisms of acyclovir resistance include a reduction or complete loss of thymidine kinase activity and viral DNA polymerase being unable to bind to aciclovir triphosphate (459, 467, 468). Patients with HSK and an aciclovir-resistant infection are typically managed through the use of DNA polymerase inhibitors which operate independently of tyrosine-kinase signalling including foscarnet or cidofovir (463, 469). These are not suitable for patients with mutations impacting DNA polymerase activity however (470).

## 7 Conclusion and future perspectives

There is a tightly-regulated interplay of molecular mechanisms which co-ordinate the cell-mediated response to HSV-1 infection and prevent the development of HSE. Several different mechanisms of immune dysregulation including stress, immunosuppressive drugs, TLR3 deficiency and LTα (180, 240, 278, 315, 338) may each be important in altering the activation and/or effector functions of T cells in response to neuro-invasion of HSV-1 resulting in poorer clinical outcomes overall. Nonetheless, there are substantial gaps in our understanding of the pathogenesis of HSE. Fragmentary evidence exists for viral entrance into the central nervous system, the immunological basis of the establishment of latency, and triggering and mechanisms of reactivation (471). Underlying mechanisms explaining how cell injury arises in HSE itself is becoming clearer with successive *in vitro, in vivo*, and clinical studies, but the relative contributions of genetic factors, viral cytopathy and immune pathology are still unclear.

Since the development of aciclovir there have been no further developments towards a more effective treatment for HSE, with increased speed of aciclovir initiation following admission being the only intervention demonstrated to improve prognosis of these patients (32). However, even following intervention with aciclovir, mortality associated with HSE remains 20%, and a further 50-70% of individuals who survive are left with neurological sequelae (32). Aciclovir halts viral replication but cannot control inflammation or eradicate latent infection (472). As a result, interest has grown in therapeutics targeting the host immune response (259). Pending results from initial trials of corticosteroids as an adjunctive treatment, the use of more targeted agents, aimed at modulating specific cytokine pathways, should be considered (387, 390, 407, 408). Immunomodulatory drugs should also be used with caution to ensure they are administered at the optimal time to reduce inflammation while still allowing for a sufficient cell-mediated immune response to be initiated (32). Vaccinations against HSV-1 are in development but are challenging to design and implement (449).

The cell-mediated adaptive immune response is at the crux of all these questions, and future studies should focus on elucidating clearly the critical and contributory factors determining the natural history of the disease, using *in vitro* and *in vivo* modelling, and the samples from patients recruited through clinical studies. Translational approaches, together with repurposing of existing targeted small molecule and biological therapies, can then be deployed to modulate deleterious immune responses and improve outcome.
